# BeeCam-AprilTag: timing the flight duration of individual honey bees in remote field settings with AprilTag and Raspberry Pi

**DOI:** 10.1016/j.mex.2025.103624

**Published:** 2025-09-14

**Authors:** Sarabeth Brandt, Diego Penaloza-Aponte, Robyn M. Underwood, Selina Bruckner, Benedict DeMoras, Margarita M. López-Uribe, Julio Urbina

**Affiliations:** a School of Electrical Engineering and Computer Science, The Pennsylvania State University, University Park, 16802, PA, USA; b Department of Entomology, The Pennsylvania State University, University Park, 16802, PA, USA; c Department of Entomology and Plant Pathology, Auburn University, Auburn, 36849, AL, USA; d Department of Entomology, Cornell University, Ithaca, NY 14853, USA

**Keywords:** Remote sensing, Open source, Data processing, Convergence research, Insect pollinators, Fiducial tags, Raspberry Pi

## Abstract

Understanding the behavior of social species that function as superorganisms requires first examining the behavior of individual members of the colony. We have developed a Raspberry Pi (RPi) and AprilTag-based camera system to record the trip durations of individual honey bees as they exit and re-enter the hive, enabling data collection of hundreds of individuals simultaneously in the context of their environment. This article details the real-time image processing capabilities of our system and the post-processing methods used to determine trip durations. AprilTag detections are collected in real-time as lines in a text file. The trip durations are obtained through a post-processing method that leverages observed honey bee behavior at the hive entrance to identify exits from and re-entries into the hive. For each AprilTag ID, detections are grouped into events. Trips are identified as consecutive “exit” and “enter” events.

## Graphical abstract

**Figure:**
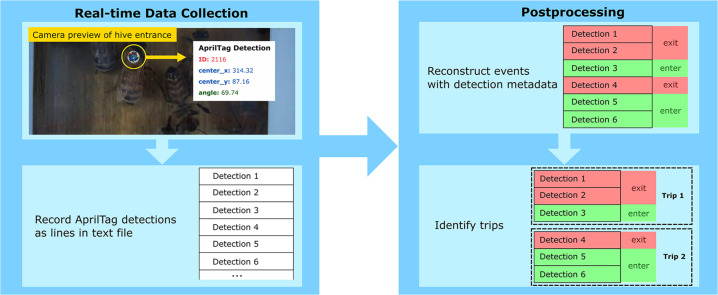

**Specifications table**

**Table:** 

**Subject area**	Engineering
**More specific subject area**	Electrical Engineering, Computer Science, Biological and Agricultural Science
**Name of your method**	BeeCam-AprilTag
**Name and reference of original method**	Automated entrance monitoring of managed bumble bees [1]
**Resource availability**	Hardware: https://doi.org/10.1016/j.ohx.2024.e00609 Zenodo Repository: https://doi.org/10.5281/zenodo.13227905

## Background

Honey bees are heavily relied upon as managed pollinators for agricultural crops across the U.S., but they have experienced high winter mortalities over the last few decades [[2], [3], [4]]. Many factors contribute to this decline, necessitating research into how honey bee colonies respond to a wide variety of stressors [5]. One of the biggest challenges of studying honey bees is the sheer number of individuals in a colony making it difficult to observe their behavior at the same time. An automated method for tracking the behavior of thousands of individual bees can enable new research into the complex behavior of honey bee colonies. Fiducial tags are markers that are easily identified by computer vision and can provide individual identification to honey bees. They have shown promise as a method to study the behavior of individual insects [[6], [7], [8], [9]]. One such example uses a Raspberry Pi (RPi) camera and AprilTag fiducial tags to automatically monitor bumble bees at their hive entrance [1]. AprilTags are printed on paper and attached to the bumble bee’s thorax, providing a unique ID number to track individuals as they enter and exit the hive. The RPi camera system acts as a motion detector to record all events at the entrance, and the AprilTags are detected from the footage in post-processing.

In order to adapt this method for honey bee colonies in remote field settings, we implement the AprilTag detection in real-time and store the detections as lines in text files. We chose AprilTags over other fiducial tags for the combination of their good detectability at small sizes, fast detection algorithm, and robustness against false positives [10]. The ability to generate custom tag families allows us to tune for these attributes, which are necessary for real-time detection with limited computational resources. The circular shape of the custom tags is also more spatially efficient on the honey bee thorax. The original method for monitoring bumble bees is designed for an indoor lab setting, which limits its applicability for a field setting. Honey bee colonies in lab settings are generally smaller than full production colonies in the field, which limits the applicability of lab results. Labs also generally have access to power, internet, fast computers, and large data storage, which are often unavailable in remote field locations. We collected data from six different apiaries in remote areas across the states of Pennsylvania and New York (USA), meaning data could only realistically be collected every two weeks. Collecting detections as text file lines consumes significantly less digital storage than image data. Additionally, honey bee colonies are larger and more active than bumble bee colonies. There is near-constant activity at the hive entrance, so motion detection wouldn’t selectively capture events. Rather than capture images of events at the entrance, we reconstruct events in post-processing using the information collected from real-time AprilTag detections.

## Method details

**Fig. 1:**
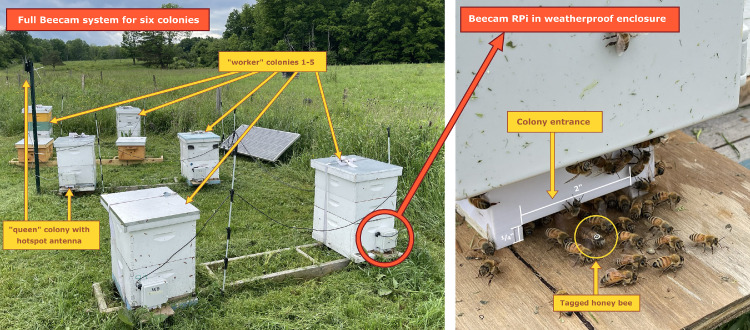
The photo on the left depicts one full Beecam system consisting of six colonies, each with a Raspberry Pi monitoring its entrance. All RPis are powered by one 12 V lithium iron phosphate battery, which is charged via solar panel [11]. The photo on the right shows the typical traffic at one hive entrance. A tagged bee walks underneath the RPi camera. While we want the bees to walk on the wooden entrance floor, many inevitably walk on the entrance ceiling instead, obscuring potential tag detections.

The full Beecam system is comprised of six Raspberry Pis, one “queen” and five numbered “workers” (W1, W2, etc.) (Fig. 1). The hardware and implementation of the full system is described in the companion HardwareX paper [11]. Here, we describe the methods in software for data collection and post-processing for one Raspberry Pi. All material for the full system setup can be found in the Zenodo repository, including the full software repository via GitHub. The real-time data collection software is included in the BeeCam Raspbian OS image. We provide all post-processing functions in the script “beecam_postprocessing.py”.

### Before data collection

It is highly recommended to plan AprilTag marking sessions ahead of time and log all ID numbers deployed throughout the season in a spreadsheet. Each entry should contain the range of ID, the location and date they were deployed, and the colony the tagged bees originate from. While not required for determining trip length, keeping track of deployed ID numbers and their assignment times allows for better filtering of false detections. For a false detection to be included in the final events and trips, it would have to contain a valid ID number (we only deployed 7000 unique IDs out of a possible 8959) and occur within a valid time frame based on the ID’s date of deployment. Our custom AprilTag family consists of over 8000 unique ID numbers, with each colony assigned a range of 1000 IDs. For example, we used tags 1000–1999 for bees from the W1 colony, 2000–2999 for the W2 colony, and so on. Bees were tagged in increments of 100 per marking session. In the first session, W1 and W2 bees received tags 1000–1099 and 2000–2099, respectively. The second session used tags 1100–1199 and 2100–2199, and this pattern continued throughout the season. This approach ensured that no two individual bees have the same ID at a given location and that each ID’s colony of origin and age can be easily determined.

Logging each session into a spreadsheet also ensures that complications in the plan are handled consistently. It was not uncommon for a colony to be too weak to tag early in the season, so we had a record to know which ID numbers were planned but never used. This log can be later used in the post-processing step to filter false detections.

### AprilTag

**Fig. 2:**
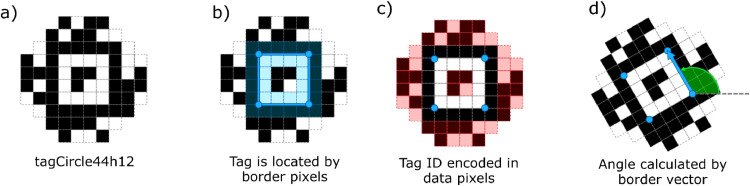
ID and spatial information contained in (a)an example tag from the custom tagCircle44h12 family. The AprilTag detection algorithm initially locates the tag by (b)the black and white border pixels. The tag is then identified by (c)the unique ID number encoded in the remaining pixels. We then calculate (d)the angle of tag using the border in order to determine the orientation of the tag.

For an individual honey bee, the duration of a single trip is obtained by the time between two events: exiting the hive and reentering the hive. Without video recording to identify these events visually, we must reconstruct these events using the spatial information from each AprilTag detection and the date/time from the full system’s external clock. Fig. 2 illustrates the information obtained from the AprilTag.

**Fig. 3:**
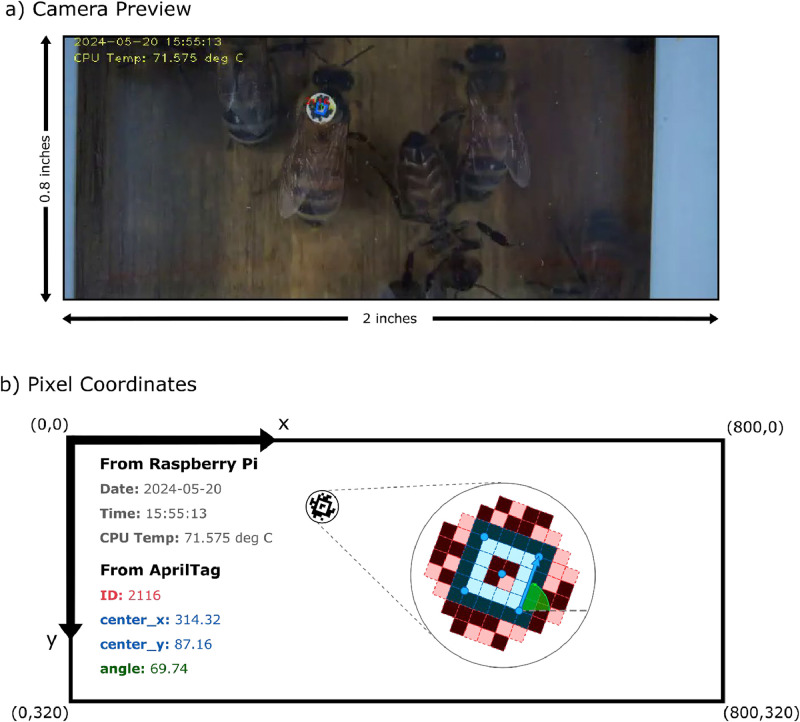
AprilTag detection in one video frame. The camera preview, shown in (a), becomes visible on the RPi desktop when the Beecam application launches. The data recorded from this frame in real-time is illustrated in (b), where the spatial information from the AprilTag and the date/time from the RPi external clock are recorded in text form instead of as an image.

An AprilTag is located first by its black and white borders, then the ID number is read from the tag’s data pixels [10]. The orientation of the tag can be quickly calculated with the border’s corner positions because we don’t need to consider out-of-plane rotation to determine if a bee is entering or exiting. When a tagged honey bee travels through the hive entrance, the AprilTag provides the individual ID, position, and orientation of the bee for each frame that the tag is successfully detected (Fig. 3).

Thus, the position and orientation of a tagged individual can be saved in one line of text instead of a much larger image file. When implemented in real-time on video frames from the camera feed, we can collect sufficient spatial and temporal information to reconstruct exit and entrance events in post-processing.

### Real-time apriltag detection

The real-time detection script “beecam_preview.py” executes automatically when the RPi boots. While the script runs, the camera is active, but no video is recorded. A preview of the camera view will become visible on the Raspberry Pi desktop once the script is active. This preview displays the date, time, and CPU temperature of the RPi. The CPU temperature is not used in any post-processing steps outlined in this manuscript, but it is important to monitor throughout the summer heat.

**Fig. 4:**
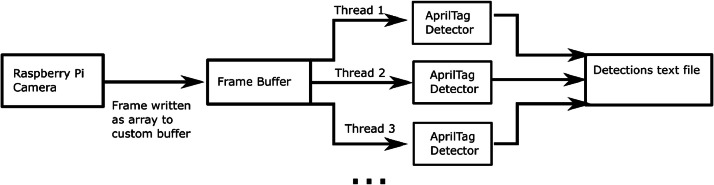
Real-time AprilTag detection. Each video frame is written as an array to a buffer which distributes them to AprilTag detector threads. Each thread applies the AprilTag detection algorithm to its frame. When a tag is detected, the data is recorded as one line into a text file.

Our script primarily utilizes the picamera2, AprilTag, and OpenCV python libraries. Fig. 4 illustrates the real-time AprilTag detection loop. The real-time detections steps are summarized as follows:

1. Write the current video frame into thread buffer.
2. The buffer sends the frame to an idle detection thread, then instructs the thread to run.
3. The detection thread applies the AprilTag detector to the frame. If any tags are detected, the thread writes the detection to the text file and highlights the detection for the preview.
4. Display the frame for the camera preview and wait for the next frame.

The picamera2 library allows functions to be injected into the camera’s main event loop. In other words, simple functions can be easily applied to every video frame. We use this functionality to write every frame into a buffer that distributes each frame to the AprilTag detector threads. Each thread searches the frame for AprilTags, records any detections, then displays the frame in the preview window with any detections highlighted. The detection algorithm is provided by the AprilTag python library.

If there is no available detector thread when the buffer attempts to send a frame to be processed, the frame is skipped. Thus, while the camera may run at a specified framerate, the true detection framerate achieved depends on the speed of the detectors. Detection framerate can be improved by reducing the resolution that the camera operates at, but reducing the resolution makes it more difficult for the small AprilTags to be detected. We achieved a rate of about 15 frames per second with a resolution of 800 × 320 pixels.

**Fig. 5:**
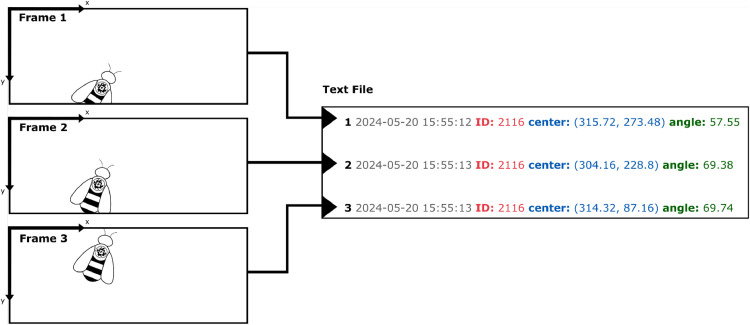
The camera captures a tagged bee entering the hive. Rather than saving the video, the date and time along with the location and orientation of the AprilTag are recorded for each video frame. This data is sufficient to reconstruct this entrance event in post-processing.

**Fig. 6:**
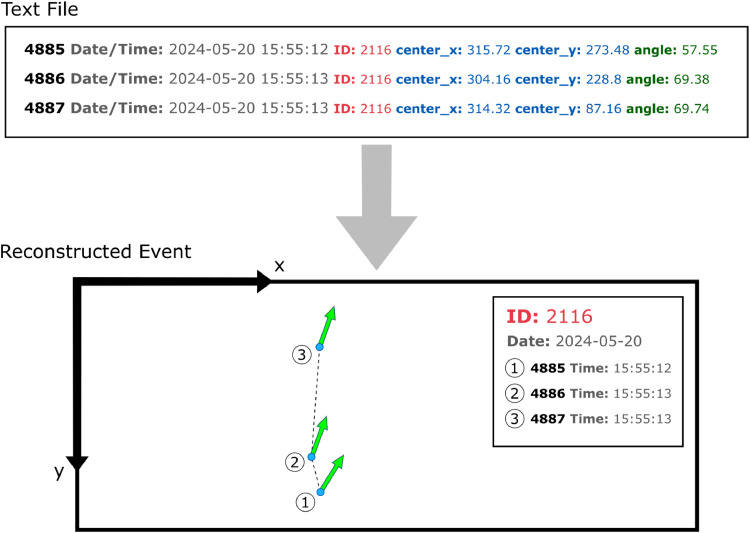
Detections from the text file are used to reconstruct the entrance event. The date and time are used to sort all detections into distinct events. Then, the spatial information from the AprilTag is used to reconstruct and classify the event.

**Fig. 7:**
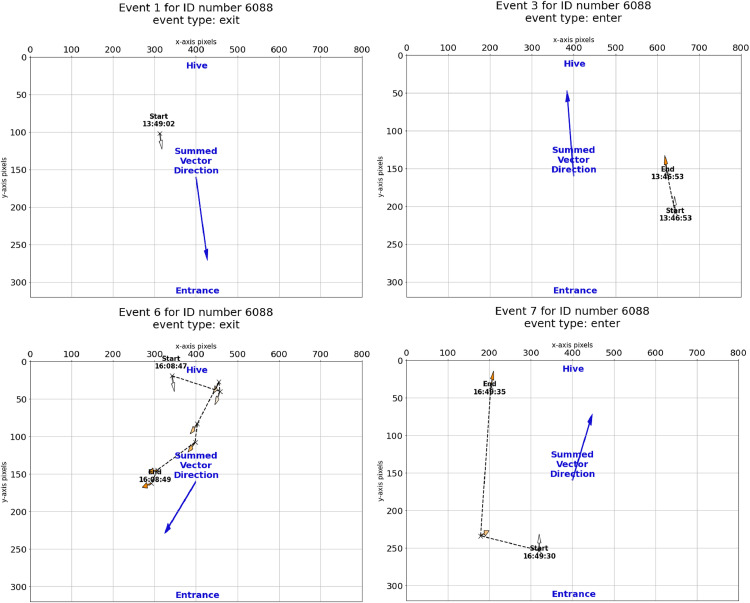
Examples of reconstructed event paths for ID number 6088. Most events are similar to Event 1 or Event 2, containing only one or two detections. For events with many detections, the path can be reconstructed. This may provide helpful context for unknown events.

Each detection is stored as a line in the text file (Fig. 5). The line also includes the date, time, and CPU temperature. Each colony cumulatively collects detections into a single text file, which is then periodically collected from the field. The file name is “[RPi hostname]_detections.txt” to keep track of which data originates from which colony. While the systems have no internet access, the RPi allows for remote connection and file transfer via SSH (terminal connection) or VNC (remote desktop connection).

### Post-processing

This post-processing code is based primarily in the Pandas library to extract trips from the text file while preserving the metadata in a multi-indexed DataFrame. Each post-processing step described here is a single function written with the intention to be straightforward to the user while being easily adjusted or added on to in the future. The steps are summarized as follows:

1. Filter out false detections
2. Group detections by ID number and sort by date/time
3. For each ID, group detections into events
4. Classify events as “exit” or “enter”
5. Pair events into trips

Note that each raw text file contains detections from a single colony. For processing multiple colonies, we recommend creating a dictionary of individual DataFrames, which can then be concatenated if needed. It is not recommended to splice the raw text files together for processing, as this will omit important context. For example, an ID number in the 2000s may have been assigned to the W2 colony, but that individual may enter a different colony nearby, for example, colony W3. In theory, this would result in four events: the bee exits W2, enters W3, exits W3, then enters W2. If the data from each colony is processed separately, the correct trip length will be found, starting with the W2 exit and ending with the W2 entrance. If this data is processed as one, an incorrect trip will be found, starting with the same exit but ending with the entrance into W3.

1. Data conversion and filtering

The function *text2df* converts this text file into a Pandas DataFrame object, where each row is a detection and each column represents the metadata (ID, position, orientation, etc.). Working with the data in this format allows for easy extraction of relevant data points for most users while preserving the raw metadata for possible further analysis.

The tagging log is also converted to a DataFrame with the function *get_tagging_log.*

The function *filter_detections* takes this DataFrame and a tagging log as inputs and removes detections that contain either an ID number outside of the logged range or a date before the very first tagging session. Detections with unused ID numbers are simply false detections, whereas detections with an impossible date usually occur if there’s an issue or delay with the external clock sync.

2. Identify events

In order to identify trips within the detections, all detections must be sorted into distinct events. A single detection represents one frame where the tag is visible to the camera, so any one event can be comprised of one or more detections. Intuitively, one might want to choose a maximum event duration based on the observed honey bee walking speed. However, due to the previously discussed fast pace of the honey bee and limitations in tag detectability, we expect many events to only be captured in a single detection. Thus, detections are instead sorted into events by a minimum time between events.

The function *get_events* takes the detections DataFrame and two user-defined variables, *minimum_time_delta* and *angle_threshold*, to generate a new DataFrame with a multi-level index for ID, event, and detection. Three new columns are included for event type, detection *time_delta*, and event summed vector angle. This function performs the following steps:

1. Group all detections by ID number and sort chronologically.
2. For each ID number, calculate the *time_delta* for all its detections, that is the duration between a detection and the one before it. The first detection will not have a *time_delta*, but it is assigned at the first detection for the first event.
3. If the next detection’s *time_delta* is less than the minimum, it is included in the same event. If it is greater than the minimum, it is assigned as the first detection of a new event. Repeat for all detections.
4. For each event, calculate the summed vector angle of all its detections.
5. Classify each event as “exit”, “enter”, or “unknown” based on the *sum_angle* and the *angle_threshhold*
6. Repeat steps 2–5 for all ID numbers.

The DataFrame allows its contents to be grouped by any specified parameters, which means detections can be easily sorted by ID to perform operations only on detections that share an ID. After detections are grouped by ID number, the *time_delta* is calculated by copying the *DT* column, shifting the copy’s contents down by 1, and subtracting the shifted copy from the original *DT* column. The result is stored in a new *time_delta* column.

Detections are sorted into events by a cumulative count of every *time_delta* entry that is greater than the minimum. This count functions as the event index for each ID number. The DataFrame can then be grouped by ID number and event number to classify individual events.

3. Classify events

Once identified, the function *get_events* classifies each event as “exit”, “enter”, or “unknown”. The fast pace and dense traffic of honey bees at the hive entrance mean that these events are consistent. While checking the camera feed in the field, we observed that a honey bee entering or exiting the hive will almost always walk in a straight line around 90 or 270 degrees, so events can be classified by the vector sum angle of its detections. All events that fit within a user-defined threshold surrounding 90 or 270 degrees are classified as “enter” or “exit” respectfully. All events that fall outside of this threshold are classified as “unknown”.

We only used the summed vector direction to classify events because, for all events, the mode for number of detections per event is 1. However, the positional information from each detection can be used to reconstruct paths in events as well (Fig. 6, Fig. 7). This is useful for further analysis of “unknown” events.

4. Identify trips

Finally, individual trips can be identified by consecutive “exit” and “enter” events for each ID number. While all detections are included in an event, not all events are included in a trip, so this function filters all detections that do not belong to a trip.

The function *get_trips* takes the events DataFrame and a *maximum_trip_duration* as inputs. All trips longer than the maximum duration are filtered out. The output is multi-indexed DataFrame with levels for ID, trip, event, and detection. New columns are added for the trip start, end, and duration. The start is determined by the time of the last exit detection, and the end is determined by the time of the first enter detection.

The function *trips_csv* provides a summary spreadsheet of all trips. If a tagging log is provided, the number of days since the individual bee was tagged is included.

## Method validation

We collected data from six different apiaries in the states of New York and Pennsylvania (USA) between April and October 2024 to test our data processing methods. Foraging trips are of particular interest for this experiment, but we cannot yet distinguish specific behaviors from this data alone. The goal of this data collection was not to draw conclusions about honey bee behavior from the identified trips but rather to collect a large sample of trip durations from the detections recorded from the AprilTags on individuals. Therefore, this section focuses on validating the assumptions made for this post-processing method based on known and expected trends in honey bee behavior and colony activity. We analyze data collected from all six colonies in Spring Mills, PA, which comprises 10,534 trips from 7498 tagged honey bees.

**Fig. 8:**
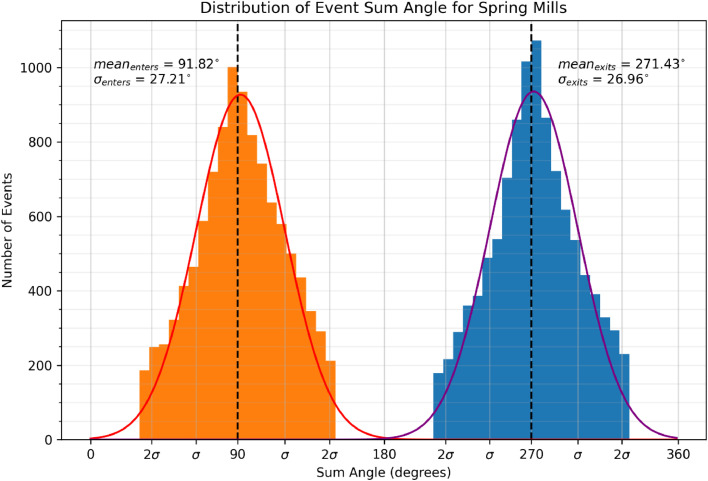
Distribution of event sum angle for all events included in trips for an angle_threshold of 60 degrees, minimum_time_delta of 60 s, and a maximum_trip_duration of 2 h.

We assume that honey bees almost always walk in a straight line in or out of the hive. In other words, we expect the sum angle of each “exit” and “enter” event to be 270 and 90 degrees respectively. Fig. 8 shows the distribution of the sum angle for each event. Both “enter” and “exit” events follow a normal distribution about their expected values. In this experiment, we choose a *minimum_time_delta* of 60 s as the shortest trip length of interest. This means that shorter trips are missed, such as guard bees exiting and quickly reentering the hive, bees taking short orientation flights, etc. These fast trips are likely processed as a single “unknown” event because the expected sum angle of a bee exiting and re-entering the hive is 0 or 180 degrees. Choosing a smaller *minimum_time_delta* thus has minimal impact on the statistical trends of trips over 60 s, but this parameter can be tuned for more specific behavioral contexts if desired.

**Fig. 9:**
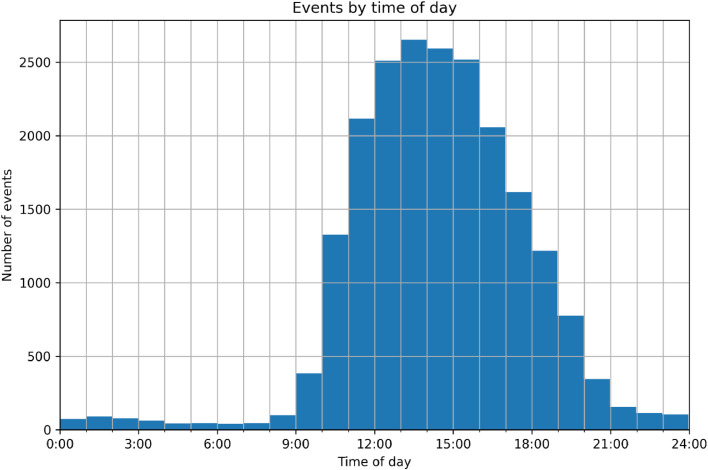
All events included in trips by time of day. Activity peaks between 13:00 and 14:00, corresponding with solar noon in the summer months.

Fig. 9 shows the distributions of all events by time of day, which supports expectations that honey bees are most active during the peak hours of sunlight. During the summer months, this occurs at noon and early afternoon hours. The time of day for each event aligns with expected hours of peak activity, that being the hours of peak sunlight. A small amount of activity is also expected when the sun is down, as bees may ventilate the hive or rest under the camera [12]. Some resting or slower activity is suggested by the number of detections per event. The percentage of all events with 10 or more detections is 9.5 %. For events occurring before 9:00 and after 21:00, this increases to 33 %. The small LED lights inside the hive entrance tunnel were active throughout the night as well, which may also contribute to the small amount of activity during these hours.

**Fig. 10:**
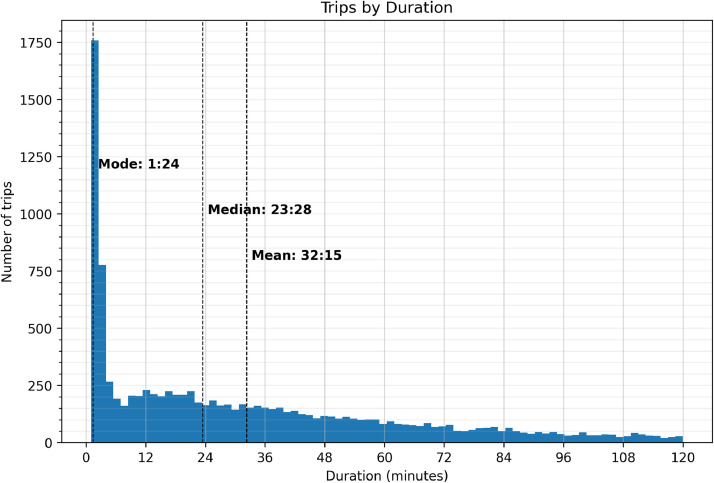
Distribution of trip duration for all colonies in the Spring Mills apiary.

Finally, we look at the distribution of trips by their duration in Fig. 10. Our results here align with what has previously been recorded, where the majority of trips occur between 1 and 4 min [7]. Additionally, foraging resources at this site (Spring Mills) were located close to the colonies, so shorter trip durations were expected.

## Limitations

One limitation of this method is the low camera resolution required for real-time detection, which makes tags more difficult to detect and increases the likelihood of missed detections. The lack of video footage to compare to the detections makes it difficult to verify any discrete trip duration. This method is best suited for monitoring very large numbers of individual insects, as we validate the dataset with known statistical trends. Additionally, this method is specifically designed for honey bees and may not be applicable to other social insect species. The assumptions underlying this method are based on observed honey bee behavior at the hive entrance. For example, a slower or more meandering insect may require a more complex approach for event identification.

**Related research article**

***For a published article:***

D. Penaloza-Aponte, S. Brandt, E. Dent, R.M. Underwood, B. DeMoras, S. Bruckner, M.M. López-Uribe, J.V. Urbina “Automated entrance monitoring to investigate honey bee foraging trips using open-source wireless platform and fiducial tags,” *HardwareX*, vol. 20, p. e00609, Dec. 2024, https://doi.org/10.1016/j.ohx.2024.e00609.

## Ethics statements

Not Applicable

## CRediT authorship contribution statement

**Sarabeth Brandt:** Writing – original draft, Visualization, Software, Methodology, Data curation, Formal analysis, Conceptualization. **Diego Penaloza-Aponte:** Writing – review & editing, Methodology, Software, Formal analysis, Resources. **Robyn M. Underwood:** Validation, Resources, Project administration, Investigation, Funding acquisition. **Selina Bruckner:** Validation, Resources, Investigation. **Benedict DeMoras:** Writing – review & editing, Validation, Resources, Investigation. **Margarita M. López-Uribe:** Writing – review & editing, Funding acquisition, Supervision. **Julio Urbina:** Writing – review & editing, Funding acquisition, Supervision.

## Declaration of competing interest

The authors declare that they have no known competing financial interests or personal relationships that could have appeared to influence the work reported in this paper.

## Data Availability

Data will be made available on request.

## References

[1] Du J., Brothers Z., Valdes L., Napp N., Petersen K. (2022). Automated entrance monitoring of managed bumble bees. Artif. Life Robot..

[2] Papa G. (2022). The Honey Bee apis mellifera: an insect at the interface between Human and ecosystem health. Biol. (Basel).

[3] vanEngelsdorp D., Meixner M.D. (2010). A historical review of managed honey bee populations in Europe and the United States and the factors that may affect them. J. Invertebr. Pathol..

[4] Wagner D.L., Grames E.M., Forister M.L., Berenbaum M.R., Stopak D. (2021). Insect decline in the Anthropocene: death by a thousand cuts. Proc. Natl. Acad. Sci..

[5] Goulson D., Nicholls E., Botías C., Rotheray E.L. (2015). Bee declines driven by combined stress from parasites, pesticides, and lack of flowers. Science.

[6] Gernat T., Rao V.D., Middendorf M., Dankowicz H., Goldenfeld N., Robinson G.E. (2018). Automated monitoring of behavior reveals bursty interaction patterns and rapid spreading dynamics in honeybee social networks. Proc. Natl. Acad. Sci.

[7] Chen C., Yang E.-C., Jiang J.-A., Lin T.-T. (2012). An imaging system for monitoring the in-and-out activity of honey bees. Comput. Electron. Agric..

[8] Boenisch F., Rosemann B., Wild B., Dormagen D., Wario F., Landgraf T. (2018). Tracking all members of a honey Bee colony over their lifetime using learned models of correspondence. Front. Robot. AI.

[9] Crall J.D., Gravish N., Mountcastle A.M., Combes S.A. (2015). BEEtag: a low-cost, image-based tracking system for the study of animal behavior and locomotion. PLOS ONE.

[10] Krogius M., Haggenmiller A., Olson E. (2019). 2019 IEEE/RSJ International Conference on Intelligent Robots and Systems (IROS), November.

[11] Penaloza-Aponte D. (2024). Automated entrance monitoring to investigate honey bee foraging trips using open-source wireless platform and fiducial tags. HardwareX.

[12] Moore D., Angel J.E., Cheeseman I.M., Fahrbach S.E., Robinson G.E. (1998). Timekeeping in the honey bee colony: integration of circadian rhythms and division of labor. Behav. Ecol. Sociobiol..

